# Risk Factors and Outcomes in Critically Ill Patients with Hematological Malignancies Complicated by Hospital-Acquired Infections

**DOI:** 10.3390/medicina59020214

**Published:** 2023-01-22

**Authors:** Adina Yerzhan, Madina Razbekova, Yevgeniy Merenkov, Makhira Khudaibergenova, Yerkin Abdildin, Antonio Sarria-Santamera, Dmitriy Viderman

**Affiliations:** 1 School of Medicine, Nazarbayev University School of Medicine, 53 Kabanbay Batyr Ave., Astana 010000, Kazakhstan; 2 Department of Anesthesiology, Intensive Care and Pain Medicine, National Research Oncology Center, Astana 010000, Kazakhstan; 3 School of Engineering and Digital Sciences, Nazarbayev University, Astana 010000, Kazakhstan

**Keywords:** hematologic malignancies, hospital-acquired infections, sepsis, febrile neutropenia, chronic hepatitis, intensive care unit, mortality

## Abstract

*Background and objectives*: Patients admitted to the intensive care unit (ICU) have an increased risk of hospital-acquired infection (HAI). A diagnosis of cancer alone increases the risk of sepsis three–five-fold, which further increases the risk of nosocomial infection, subsequently deteriorates results, and leads to high mortality. In this study, we aimed to assess the mortality rate among hematologic oncologic patients with suspected infection who were subsequently admitted to the ICU and the predictive factors that are associated with high ICU mortality. *Materials and Methods*: This retrospective cohort study was conducted in the hematological oncology critical care unit of a tertiary care hospital between November 2017 and February 2021. We analyzed anonymized medical records of hospitalized hematologic oncologic patients who were suspected or proven to have infection in the hematology-oncology department and were subsequently transferred to the ICU. *Results*: Both shorter hospitalization and shorter ICU stay length were observed in survivors [9.2 (7.7–10.4)] vs. non-survivors [10 (9.1–12.9), *p* = 0.004]. Sepsis had the highest hazard ratio (7.38) among all other factors, as patients with sepsis had higher mortality rates (98% among ICU non-survivors and 57% among ICU survivors) than those who had febrile neutropenia. *Conclusions*: The overall ICU mortality in patients with hematologic malignancies was 66%. Sepsis had the highest hazard ratio among all other predictive factors, as patients with sepsis had higher mortality rates than those who had febrile neutropenia. Chronic hepatitis (HBV and HCV) was significantly associated with higher ICU mortality.

## 1. Introduction

According to the WHO, in 2017 there were 11 million sepsis-related deaths worldwide, which comprised 20% of all deaths. Sepsis can develop in both community and healthcare settings, but in the latter, it is more frequently associated with poor outcomes. Its severity is because hospital-acquired infections (HAIs) are often resistant to antibiotics and can deteriorate already sick patients with other comorbidities and diagnoses.

There are considerable disparities in the incidence of sepsis, but about 85% of all sepsis cases are in low- and middle-income countries. Consequently, studying sepsis incidence and the burden of HAI on a specific cohort of patients has special importance in health care settings within the middle-income country Kazakhstan [1].

Sepsis is “defined as a life-threatening organ dysfunction caused by the dysregulation of a host’s response to infection” [2,3]. In turn, the definition of febrile neutropenia is a one-time oral temperature higher than 38.3 °C, or an oral temperature sustained for more than 1 h of 38 °C or higher in patients with an absolute neutrophil count less than 500 cells/μL (or expected within 48 h to decrease to less than 500 cells/μL) [4]. Some studies suggest the interchangeable usage of the terms “neutropenic sepsis” and “febrile neutropenia”, yet the terms “sepsis” and “febrile neutropenia” cannot be used interchangeably [5]. A study conducted using data from hematologic patients showed that septic patients with neutropenia had worse outcomes in comparison with non-neutropenic patients [6]. The dysregulation of neutrophil recruitment is correlated with sepsis, but further studies are needed so that neutrophilic status criteria can be included within the diagnosis criteria and prognosis of sepsis [7,8,9,10,11].

The overall mortality rate in the population of patients with oncologic hematologic malignancies is higher than for other patient populations. They tend to have long hospital stay lengths, which subsequently increases HAI risks, and they often require ICU admission due to treatment-related complications, infections, and organ failures. ICU mortality remains high for this population of patients with one study reporting it to be from 45.6% to 57%, with even further increases with longer term survival [12,13]. In this study, we aimed to assess the mortality rate among hematologic oncologic patients with suspected infection who were subsequently admitted to the ICU as well as what predictive factors were associated with high ICU mortality rates at the tertiary hospital in Kazakhstan. 

## 2. Materials and Methods

### 2.1. Patient Cohort

After approval from the Nazarbayev University School of Medicine (NUSOM) Institutional Review Board and the Local Ethics Committee of the National Research Oncology Center (NROC), we conducted a retrospective cohort study. It took place within the hematological oncology critical care unit of the tertiary care hospital between November 2017 and February 2021. We analyzed anonymized medical records of hospitalized hematologic oncologic patients who were suspected or proven to have an infection in the hematology-oncology department and subsequently were transferred to the ICU. The inclusion criteria were being 18 years and older; having diagnosed hematological malignancies; having suspected or confirmed infection (to have at least one of these three: febrile neutropenia, sepsis, or septic shock); and being treated in ICU. The exclusion criteria were having a not clearly described study design, patient clinical data, missing sepsis criteria or study outcomes; pediatric patients (younger than 18 years old). Patients were receiving chemotherapy, immunosuppressive therapy, and, in some cases, had bone marrow transplantation. The diagnostic criteria used for the diagnosis of the patients used in this study were taken from the national clinical protocol for diagnosis and treatment of febrile neutropenia developed in 2015 and were as follows [14]: febrile neutropenia—body temperature higher than 38 °C for more than 1 h with an absolute neutrophil count less than 500 cells/μL (or expected to decrease to less than 500 cells/μL in the upcoming 48 h); sepsis—SIRS criteria and infection (diagnosed or suspected); severe sepsis—sepsis and dysfunction of one or more organs; septic shock—severe sepsis and hypotension (systolic blood pressure <90 mm Hg, mean arterial blood pressure <70 mm Hg with systolic arterial pressure decrease for more than 40 mm Hg) despite 1 h of adequate infusion and having serum lactate level >4 mmol/L.

### 2.2. Data Collection

Personal identifiers were deleted before release of the data. We extracted data on age, gender, primary diagnosis, co-morbidities, date of admission to the ICU, length of ICU and hospital stays, length of days between hospitalization and sepsis development, discharge status (survived or died), sepsis status, septic shock status, febrile neutropenia status, pneumonia status, bone marrow transplantation status, laboratory markers (hemoglobin, white blood cells, platelets, red blood cells), and inoculated infection causing microorganisms and antibiotics.

### 2.3. Statistical Analysis

We used mean and standard deviation (SD) or median and interquartile range (IQR) for continuous variables. Frequency and percentages were used for categorical data. We used Student’s *t*-test to compare continuous variables between the survivors and non-survivors, while chi-square test was used for categorical variables. We used Cox proportional hazards (PH) regression model to examine the relationship between independent variables and mortality in which hazard ratios (HRs) were used to quantify the associations.

For all analyses, significance was set at *p*-value < 0.05 (two-tailed). The statistical analysis was performed using STATA V.14.0 software (StataCorp).

## 3. Results

### 3.1. General Demographic 

In total, 61 patients from the hematology and hematologic stem cell transplant unit were admitted to the ICU during the period of study (Table 1). Of these, there were 19 (31.15%) females and 42 (68.85%) males. The median age was 35 [interquartile range (IQR), 27–51], with a range from 19 to 70 years. The average length of hospital stay was 31.64 ± 16.3 (95% CI 27.4–35.8), the average length of ICU stay was 14.87 ± 11.3 (95% CI 11.9–17.7), and the average days before sepsis developed was 17.76 ± 14.5 (95% CI 14–21.5). The hazard ratio of age when adjusted for types of transplants (allogenic, autogenic) was found to be 1.064 [95% CI 1.02–1.2], *p* = 0.001 < 0.05; hence, it seems that each yearwas associated with 1.06 increase in the risk of not surviving ICU when adjusted for type of bone marrow transplantation (allogenic, autogenic).

The most prevalent diagnosis among the ICU admitted patients within the hematology and transplant units was acute myeloid leukemia (39.43%). The second was acute lymphoblastic leukemia (26.23%), and non-Hodgkin’s lymphoma placed third (14.75%). Other less prevalent diagnoses were Hodgkin’s lymphoma, chronic myeloid leukemia, multiple myeloma, chronic lymphoid leukemia, aplastic anemia, and myelodysplastic syndrome.

A variety of causative agents were identified. In some cases, the microorganisms were not extracted from the culture. When causative microorganisms were extracted, the most frequently seen single microorganism causes were *Klebsiella pneumoniae* (6.5%), *Staphylococcus hominis* (6.5%), *Pseudomonas aeruginosa* (9.8%), *Enterococcus faecium* (6.5%), and *Methicillin-resistant Staphylococcus aureus* (9.8%). The least frequent were *Candida albicans* (4.9%), *Acinetobacter baumannii* (3.2%), *Staphylococcus epidermidis* (6.5%), *Staphylococcus aureus* (3.2%), and *Streptococcus pneumoniae* (1.6%). In other cases, multiple organisms were cultured, meaning that there were superinfections.

Concerning treatments, the most frequently used were penicillin (ampicillin for gram positive coverage), broad spectrum penicillin (piperacillin-tazobactam for gram negative coverage), cephalosporins (ceftriaxone and cefepime for gram negative coverage), fluoroquinolones (moxifloxacin for gram positive and ciprofloxacin for gram negative coverage), vancomycin (for MRSA coverage), sulfonamide/trimethoprim, carbapenems (imipenem for gram negative coverage), linezolid (for gram positive coverage), aminoglycoside, and colistin. Clindamycin, metronidazole, and moxifloxacin were mainly used for anaerobic coverage.

Out of 61 admitted patients, 40 deceased and 21 were discharged upon completion of treatment and gradual recovery. A total of 14 (35%) of the deceased and 5 (23.8%) of the living patients were female. The mean age of living patients was 36.4 ± 13.3 (95% CI 30.4–42.5) and it was 40.2 ± 14.9 (95% CI 35–45.3) for deceased patients. The mortality rate among hematologic oncologic patients with suspected infection who were subsequently admitted to the ICU was 65.57%.

### 3.2. Hospital/ICU Stay Length and Days before Sepsis Developed

The average hospital stay for the deceased was 31 ± 17.2 (95% CI 25.5–36.5), while the living group had an average of 32.81 ± 14.9 (95% CI 25.9–39.6). 

The average ICU stay for the deceased was 15.4 ± 11.4 (95% CI 11.7–19), while the living group had an average of 13.9 ± 11.2 (95% CI 8.67–19.2). The difference was statistically not significant (*p* = 0.68 > 0.05).

The average days before sepsis developed for the deceased was 17.6 ± 15.2 (95% CI 12.7–22.5), while the living group had an average of 18.1 ± 13.6 (95% CI 11.8–24.5).

One of the most prevalent complications during hospital stays was pneumonia (80.3%). A total of 88% of the deceased and 67% of the living had pneumonia. The difference in pneumonia between the two groups of patients was statistically significant (*p* = 0.052), and it was 1.5 times (HR = 1.51) more probable that a patient with pneumonia would not survive their stay in the ICU in comparison with those who did not have pneumonia. 

### 3.3. Sepsis, Septic Shock, and Febrile Neutropenia

A total of 98% of the deceased and 57% of the living developed sepsis; the difference between the deceased and living groups in sepsis status was statistically significant (*p* = 0.00 < 0.05). Moreover, the hazard ratio (HR) was 7.38, meaning that people who had sepsis were 7.38 times more likely to die than those who did not have sepsis. 

A total of 73% of the deceased and 5% of the living developed septic shock; the difference between the deceased and living groups in septic shock status was significant (*p* = 0.00 < 0.05). Patients who had septic shock were 2.57 times more likely to die than those who did not have septic shock (HR = 2.35). 

A total of 58% of the deceased and 76% of the living patients developed febrile neutropenia, but no significant difference was seen between the two groups (*p* = 0.149 > 0.05). 

In the population of study, sepsis was mainly caused by gram positive bacteria (36%), gram negative bacteria (29.5%), or fungi (*Candida albicans*, 4.9%). 

Among deceased patients, in 40% of the cases sepsis was due to gram positive bacteria and in 33% of cases it was due to gram negative bacteria; however, in both cases the difference between the two groups was statistically insignificant (*p* = 0.38 and *p* = 0.48 respectively, both >0.05).

### 3.4. Comorbidities (Chronic Hepatitis and Hypertension)

Moving on to comorbidities, the most encountered comorbidities were hypertension (6.6%) and chronic hepatitis status (29.5%). A total of 40% of the deceased and 10% of the living had chronic hepatitis. The difference in chronic hepatitis between the two groups of patients was statistically significant (*p* = 0.01 < 0.05). A total of 8% of the deceased and 5% of the living had chronic hypertension. The difference in hypertension between the two groups of patients was not statistically significant (*p* = 0.68 > 0.05). Even if slightly different, it was 1.6 times (HR = 1.61) more probable that a patient with hypertension would not survive their stay in the ICU in comparison with those who did not have hypertension. 

### 3.5. Bone Marrow Transplantation Status

A total of 34.4% of all patients had undergone bone marrow transplantation (BMT). A total of 45% of the deceased and 14% of the living had BMT. The difference in BMT status between the two groups of patients was statistically significant (*p* = 0.01 < 0.05). Among 19 deceased patients, 3 had received autologous and 15 allogeneic transplants, whereas all surviving patients had received autologous transplants. For the allogenic transplants, donors were first-degree relatives, and the source was peripheral blood or bone marrow. 

A total of 95% of patients who had BMT and 78% who did not receive BMT developed sepsis. Even though the difference turned out to be statistically insignificant (*p* = 0.075 > 0.05), the hazard ratio of BMT status related to the outcome of sepsis was 0.52.

### 3.6. Laboratory Values

Regarding laboratory values (Table 2), an unpaired, two-tailed *t*-test was carried out to see if there were significant differences separately for either the deceased or the living patients. This was done to see if the patients’ common blood count (CBC) values were statically different before and after admission to the ICU. 

Before admission to the ICU, the hemoglobin (*p* = 0.031 < 0.05) and platelet (*p* = 0.003 < 0.05) level averages were statistically significant, but not the white blood cell level (*p* = 0.32 > 0.05). Immediately after their ICU discharges, only platelet (*p* = 0.000 < 0.05) levels were statistically different between the surviving and non-surviving groups.

## 4. Discussion

The main finding of this work was the high mortality rate (66%) of the patients who were analyzed. Patients admitted to the ICU already have an increased risk of HAI. Diagnosis of cancer alone increases the risk of sepsis three–five-fold, further increasing the risk of nosocomial infection, subsequently deteriorating results, and raising the risk of mortality [16]. Not only was mortality risk higher for those in the ICU (33%) in comparison with those not in the ICU (8%), but also the 6-month survival rate was significantly lower for those in the ICU (47%) in comparison with non-ICU (77%) patients [17]. 

The overall mortality rate for oncologic hematologic patients who were admitted to the ICU ranged from 20% to 65% in various studies due to the nature of severe baseline diagnosis, severe complications, disease progression, and immunosuppressive therapies [18].

The early clinical diagnosis of sepsis has pivotal importance in decision-making, such as whether to take blood cultures, the initiation or review of antibacterial therapy, early fluid resuscitation, transfer to the ICU, and advanced monitoring. 

In this study, we found that living patients were younger than deceased patients; each age year was associated with a 1.04 increase in the risk of dying in the ICU. Hazard ratio increased to 6% (HR = 1.06, *p* = 0.001), even after adjusting for types of bone marrow transplantation (autogenic, allogenic), meaning that irrespective of BMT status, age increases risk of mortality. Studies regarding advanced age as a risk factor for ICU mortality have split results. The advanced age effect identified here may not alone be responsible for a poor ICU outcome, but it probably is capturing the effect of more severe causes like comorbidities, the severity of main diagnoses, and developed complications [19,20,21]. Moreover, another confounder for the higher age group’s ICU outcomes may be decreased willingness for life-sustaining outcomes, especially when life expectancy is limited [22]. 

It took longer for the survivor group to develop infection, which could be due to their comparatively better baseline health conditions and more effective immune defense against infections; however, the difference was insignificant (*p* = 0.9 > 0.05).

Interestingly, BMT status was found to be protective for sepsis development. Studies concluded that BMT patients have a higher mortality risk and longer hospital stays in comparison with non-BMT patients [23]. This could be because of conditioning regimens and delays in immune reconstruction after BMT, leading to host immune system dysfunction. This may be responsible for predisposing patients to HAI. Patients who had infections or suspected infections that require immune cell reaction were not able to combat the infections; aids for further deterioration of the condition were required. In this study too, a higher percentage of the deceased (45%) than living (14%) had BMT. 

It appears that febrile neutropenia was more prevalent among ICU survivors (76%) than non-survivors (58%), and it seemed to be a protective factor (HR = 0.58). Those who had sepsis or septic shock may have had more severe disease courses or complications as, by definition, sepsis is defined as life-threatening organ dysfunction. It is not surprising that patients with sepsis had higher hazard ratios (7.38), as patients with sepsis had higher mortality rates (98% among ICU non-survivors and 57% among ICU survivors) than those with febrile neutropenia. However, febrile neutropenia was described in studies as a risk factor for mortality and ICU admission in patients receiving chemotherapy treatment for cancer [24]. 

Chronic hepatitis is defined as inflammation of liver that lasts longer than 6 months, due to different etiologies with hepatitis B and C as the most common causes. Previously it has been described that chronic hepatitis increases the risk of hematologic cancers, particularly non-Hodgkin lymphoma [25]. Alvaro-Meca et al. found that chronic hepatitis C increases the risk of death of ICU-admitted patients with cirrhosis [26]. 

We tried to find out if chronic hepatitis (HBV and HCV) is a risk factor for ICU mortality in hematologic oncologic patients. Indeed, it was found to increase poor outcomes, and it was 2.16 times (HR = 2.16) more probable that patients with chronic hepatitis did not survive the ICU in comparison with those who did not have chronic hepatitis.

Patients who developed chronic infections were those who failed to clear HBV within 12 months, and thus had high levels of HBV replication [27]. Higher levels of HBV replication in turn were seen in patients with concomitant bacterial co-infection in comparison with those who had a lower level of HVB replication [28]. Consequently, we hypothesized that co-infections with other microorganisms weakens already exhausted oncologic hematologic patients, further increasing mortality. This might be especially true for patients with a history of long hospital stays and ICU admissions, due to the high probability of HAIs and being exposed to resistant strains of bacteria in their immunocompromised states. Another possible explanation for this association could be the fact that patients with oncologic conditions and those receiving immunosuppressive therapies, such as chemotherapy, have higher probability of HBV reactivation or hepatitis flareups. This, in consequence, can lead to the delay or cessation of treatment, thus increasing the mortality rate [29].

Recent reports from Kazakhstan have found that hospital-acquired infections and sepsis are predominantly caused by gram negative pathogens such as *K. pneumoniae*, *P. aeruginosa*, and *E. coli* [30]. The establishment of regular infection control audits, active surveillance, rational antibacterial therapy, and general hygiene measures might reduce the incidence of sepsis and sepsis-related mortality [30,31,32]. The epidemiological data on the incidence of hospital-acquired infections and sepsis in developing countries are poorly reported [30].

One of the limitations of this study is its small sample size; this limits the precise interpretation of confidence intervals and *p* values. Some variables such as length of hospital stay, febrile neutropenia, hypertension, BMT status, pneumonia, and causative microorganism (gram positive or gram negative sepsis) status had p values over 0.05, showing no clear statistically significant difference between ICU survivors and non-survivors. However, looking at respective hazard ratios, we can say that there is some evidence of an effect, but the results missed statistical significance due to a low number of subjects in each group [33]. Another limitation of studies conducted at tertiary hospital settings could potentially be referral bias (also admission bias). Patient populations at tertiary hospitals seem to be different from those in other hospital settings due to more severe illnesses and a higher prevalence of comorbidities. This renders the association between exposure and outcome less helpful.

## 5. Conclusions

The overall ICU mortality in patients with hematologic malignancies was 66%. Sepsis had the highest hazard ratio among all other predictive factors, as patients with sepsis had higher mortality rates than those who had febrile neutropenia. Chronic hepatitis (HBV and HCV) was significantly associated with higher ICU mortality.

## Figures and Tables

**Table 1:** Patient characteristics, comorbidities, and complications.

Variables	All (n = 61)	Survivors (n = 21)	Non-Survivors (n = 40)	*p*-Values	Hazard Ratio (HR) Unadjusted (95% CI)	*p*-Values	Hazard Ratio (HR) Adjusted for Age, Sex	*p*-Values
**Age**	38.8 ± 14.3	36.4 ± 13.3	40.2 ± 14.9	0.348	1.04 (1.002–1.08)	0.037		
**Gender**								
Female (Reference)	19 (31.15%)	5 (24%)	14 (35%)	0.37				
Male	42 (68.85%)	16 (76%)	26 (65%)		0.52 (0.26–1.04)	0.065		
**Hosp stay length**	31.6 ± 16.3.1	32.8 ± 14.9	31 ± 17.2	0.689	NA			
**Intensive care unit (ICU) stay length**	14.9 ± 11.3	13.9 ± 11.2	15.4 ± 11.4	0.643	0.98 (0.95–1.00)	0.057	0.98 (0.95–1.05)	0.29
**Days from hospitalization to sepsis**	17.8 ± 14.5	18.1 ± 13.6	17.6 ± 15.2	0.901	0.95 (0.93–0.98)	0.000	0.95 (0.92–0.98)	0.001
**Febrile neutropenia**								
Yes	39 (63.9%)	16 (76%)	23 (58%)		0.58 (0.30–1.12)	0.106	0.40 (0.2–0.8)	0.01
No (Reference)	22 (36.1%)	5 (24%)	17 (43%)	0.149				
**Gram positive**								
Yes	22 (36.1%)	6 (29%)	16 (40%)		1.42 (0.74–2.73)	0.290	1.29 (0.62–2.66)	0.496
No (Reference)	39 (63.9%)	15 (71%)	24 (60%)	0.377				
**Gram negative**								
Yes	18 (29.5%)	5 (24%)	13 (33%)		0.49 (0.24–1.04)	0.067	0.54 (0.25–1.18)	0.122
No (Reference)	43 (70.5%)	16 (76%)	27 (68%)	0.48				
**Hypertension (HTN)**								
Yes	4 (6.6%)	1 (5%)	3 (8%)		1.61 (0.48–5.35)	0.437	1.51 (0.44–5.15)	0.509
No (Reference)	57 (93.4%)	20 (95%)	37 (93%)	0.681				
**Pneumonia**								
Yes	49 (80.3%)	14 (67%)	35 (88%)		1.51 (0.58–3.92)	0.4	1.75 (0.56–5.47)	0.337
No (Reference)	12 (19.7%)	7 (33%)	5 (13%)	0.052				
**Chronic hepatitis**								
Yes	18 (29.5%)	2 (10%)	16 (40%)		2.16 (1.12–4.15)	0.02	2.33 (1.14–4.73)	0.02
No (Reference)	43 (70.5%)	19 (90%)	24 (60%)	0.013				
**Septic shock**								
Yes	30 (49.2%)	1 (5%)	29 (73%)		2.57 (1.27–5.18)	0.008	2.34 (1.08–5.09)	0.032
No (Reference)	31 (50.8%)	20 (95%)	11 (28%)	0.000				
**Bone marrow transplant (BMT) status**								
Yes	21 (34.4%)	3 (14%)	18 (45%)	0.016	0.71 (0.35–1.41)	0.32	0.77 (0.36–1.62)	0.489
No (Reference)	40 (65.6%)	18 (86%)	22 (55%)					
**Sepsis**								
Yes	51 (83.6%)	12 (57%)	39 (98%)		7.38 (1.01–53.9)	0.049	8.15 (1.09–60.9)	0.041
No (Reference)	10 (16.4%)	9 (43%)	1 (3%)	0.000				

**Table 2:** Laboratory data [15].

		Before			After		
**Variables**	Normal values	Survivors	Non-survivors	*p*-value	Survivors	Non-survivors	*p*-value
**Hemoglobin**	Male: 13.5–17.5 g/dL Female: 12.0–16.0 g/dL	91.6 ± 29.1	76.1 ± 24.3	0.031	73.2 ± 9.5	68.8 ± 14.6	0.209
**White blood cells**	4500–11,000/mm^3^	6.1 ± 11.4	28.7 ± 100.1	0.321	3.0 ± 1.9	7.1 ± 23.4	0.447
**Platelets**	150,000–400,000/mm^3^	145.1 ± 138.7	58.6 ± 78.9	0.003	213 ± 225	27.9 ± 30.9	0.000

## Data Availability

Dataset is available upon request.
